# Heredity of angel wing and growth performance in White Roman geese

**DOI:** 10.5713/ab.25.0037

**Published:** 2025-05-12

**Authors:** Min Jung Lin, Sheng Der Wang, Chao Hsien Lee, Shen Chang Chang, Shao Yu Peng, Tzu Tai Lee

**Affiliations:** 1 Bachelor of Program in Scientific Agriculture, National Pingtung University of Science and Technology, Pingtung, Taiwan; 2 Northern Region Branch, Taiwan Livestock Research Institute, Ministry of Agriculture, Changhua, Taiwan; 3 Dairy Assocation of Taiwan, Taipei, Taiwan; 4 Department of Animal Science, National Chiayi University, Chiayi, Taiwan; 5 Department of Animal Science, National Pingtung University of Science and Technology, Pingtung, Taiwan; 6 Department of Animal Science, National Chung Hsing University, Taichung, Taiwan; 7 The iEGG and Animal Biotechnology Center, National Chung Hsing University, Taichung, Taiwan; 8 Smart Sustainable New Agriculture Research Center (SMARTer), Taichung, Taiwan

**Keywords:** Angel Wing, Body Weight, Liability Heritability, White Roman Goose

## Abstract

**Objective:**

The purpose of the study was to investigate the liability heritability of incidence of angel wing (LHIAW) on growth and egg production performance.

**Methods:**

A total of 1,696 geese including 990 offspring from the heavy body weight line (selecting for 6 generations) and 775 offspring from the high egg production line (selecting for 3 generations), and 69 birds of their parent group of the two lines were observed of incidence of angel wing (IAW).

**Results:**

In the heavy body weight line of the White Roman geese, the IAW was 54.6%. Among 294 progenies from families, the IAW was 65.6%. The estimated LHIAW for this line was 0.39. In the high egg production line of the White Roman geese, the IAW was 42.3%. Among 124 progenies from families, the IAW was 43.5%. The estimated LHIAW for this line was 0.03. The both-side angel wing type of geese was significantly heavier on body weight than those of normal wing type and right angel wing type at 8 weeks old (4.19 vs 4.07 and 4.07 kg/bird; p = 0.0116) in the heavy body weight line. The normal wing type of geese was significantly heavier on body weight than those of left angel wing type at 14 weeks old (4.86 vs 4.69 kg/bird; p = 0.0213) in the high egg production line.

**Conclusion:**

The LHIAW in the heavy body weight line and the high egg production line were separately estimated as 0.39 and 0.03, respectively. The results imply that selection for heavy body weight may concomitantly select the gene of angel wings.

## INTRODUCTION

An anomaly in certain growing waterfowls has been aware that it is characterized by a lateral rotation of the distal end of the fore limbs, either single side or both sides, mostly at carpometacarpus, where the extremity of the wing twists outward away from body lateral [1]. The abnormal wing has been termed of slipped wing [1–3], twisted wing [4] or angel wing (AW) [5]. The swans’ wings were bandaged close to their bodies for one week, and their food intake was adjusted accordingly [6]. AWs have a high likelihood of being treated if the condition is identified early.

A genome-wide association study identified nine single nucleotide polymorphisms (SNPs) across five chromosomes that were significantly associated with the AW trait [7]. The AW is torqued outward, away from the body laterals at the carpometacarpus, and shows a slight to moderate dilated space in the carpometacarpal joint [8]. Normal wing geese have a greater angle than AW geese at the age of 14 weeks. The Whooping Crane (Grus americana) chick was unable to fully extend its left wing, which was held at an abnormal angle at the carpal joint [9]. AW is a developmental deformity of the carpometacarpal region in waterfowl and larger waterfowl, resulting in the displacement of primary remiges [10]. Field surveys showed that incidence of angel wing (IAW) ranged between 5% to 50%. The IAW of White Chinese goose had higher than White Roman goose (13.6% vs. 6.9%) [11]. Estimated 4.4% of Masked Booby (Sula dactylatra) chicks on Clipperton Island exhibited AW [12]. The total IAW was 6.67% and 8.86% in 150-day-old White Zhedong geese and 70-day-old Hybrid-Wanxi geese, respectively [13]. The AW line on severity scores and IAW had a significantly higher than those in heavy body weight line and commercial line at age of 14 weeks in White Roman goose, respectively [14]. The IAW on the left side was higher than that on the right side [13]. The severity score and IAW were lower in males than females at 6 weeks-old. There are differences in the IAW between different animal species, breed and sex [15].

High stocking density leads to overcrowding, which decreases feed intake and results in lighter body weights and poorer uniformity among the geese [16,17]. Stocking density did not significantly affect the severity score and IAW among the different treatments [17,18]. However, the severity score and IAW were higher in the angel-winged line compared to the normal-winged line and the commercial line. This AW outbreak coincided with high nestling mortality, which was apparently related to food shortage. Geese fed with high concentration nutrient diet (geese provided crude protein 20% of starter diet during 0 to 4 weeks of age and crude protein 15% of grower diet during 5 to 14 week of age) in comparison with those fed with low nutrient concentration diet (geese provided crude protein 15% of grower diet during 0 to 14 week of age). Geese fed a high concentration nutrient diet showed a tendency toward higher severity scores (1.10 vs. 0.58) and incidence rates (34.6% vs. 18.5%) compared with those fed a low concentration nutrient diet at 14 weeks of age [14]. The IAW was higher in both the T-2 toxin and antioxidant groups compared to other groups at 6 weeks of age. The stocking density of geese does not affect the severity scores of AW and IAW. However, geese fed a high-protein diet in the early stages tend to have a higher incidence of IAW. Additionally, diets supplemented with T-2 toxin and antioxidants also increase the occurrence of IAW [15].

Many studies have investigated the effects of nutrition, breed, and sex on the severity scores of AW and IAW [8,13,15,17,18]. However, IAW has a genetic component, and goose breeders often eliminate geese with AW directly. Research on the genetic aspects of IAW is relatively limited. This study aims to investigate the liability heritability (h^2^) of IAW and compare the types of AW in relation to growth and egg production performance. The findings will serve as a reference for researchers and breeding goose farms in selecting and culling based on these traits.

## MATERIALS AND METHODS

### Animals and management

The experimental protocol was approved by the Animal Care and Use Committee of Changhua Animal Propagation Station, Livestock Research Institute, Executive Yuan, Changhua, Taiwan (CH96-03; CAPS-LRI, located at 23°51′N and 120°33′E), Taiwan. Drinking water was provided *ad libitum*.

The White Roman geese used were originally imported from Denmark and USA in 1975 and 1985, respectively, and had genetically been selected into two lines, one for heavy body weight and the other for high egg production, from 1997 on at Northern Region Branch, Taiwan Livestock Research Institute, Taiwan.

The starter ration contained metabolizable energy (ME) of 2,900 kcal/kg and crude protein of 20% for birds during 0 to 4 weeks of age and the grower ration contained an ME of 2,800 kcal/kg and crude protein of 15% for birds from 5 to 14 weeks of age. Resting geese were give a restricted amount of resting rations (13.0% CP and 2,350 kcal/kg ME). Thereafter, the laying geese were fed *ad libitum*. The laying diet contained 18.0% CP and 2,700 kcal/kg ME (Table 1).

### Experimental design

A total of 1,696 geese including 990 offspring from the heavy body weight line (selecting for 6 generations) and 775 offspring from the high egg production line (selecting for 3 generations), and 69 birds of their parent group of the two lines were observed of IAW. These 69 breeding geese were selected from the existing flock (131 ganders and 358 geese) in 1994, which had excellent egg production and body weight [19]. This study geese records of either AW or normal wing conditions. In the selection process for the heavy body weight line and high egg production line, only the 8-week body weight and egg production of the first laying cycle were used as selection criteria. There was no selection or culling based on IAW in White Roman geese, geese only records of IAW were selection process.

Line of high egg production and heavy body weight in this flock were selected within a Closed colony population. Seventy-two geese hatched were used as the parental generation (G0). Geese were selectively bred for high egg production and heavy body weight lines. the heavy body weight line had undergone six generations of selection, and the high egg production line had undergone three generations. For the heavy body weight line, geese were selected based on their breeding values for body weight at 8 and 14 weeks of age. These selected geese were then placed in cages for pedigree breeding, with one generation selected each year. For the high egg production line, selection was based on the breeding values for egg production of the first laying cycle of caged geese, with pedigree breeding occurring in the second or third laying cycle. Selection for this line took place over 2–3 years per generation.

The calculation of breeding values was based on the estimation of genetic parameters using the variance component estimate version 4.2.5 software [20]. A single-trait animal model was applied, and the restricted maximum likelihood method was used to estimate the heritability of egg production in the high egg production line of White Roman geese. Following this, the breeding values for the number of eggs produced in the first laying cycle of the new generation were estimated using the best linear unbiased prediction (BLUP) method, implemented through the PEST version 4.2.3 software [21].

### The types of angel wing and incidence of angel wing

**Figure 1:**
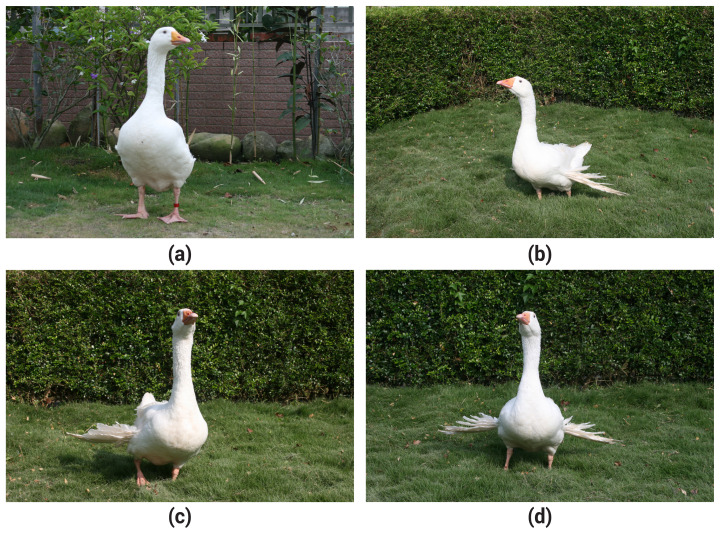
Morphological characteristics of goose wings. (a) Both-side normal wings, (b) the right-side normal wing and the left side angel wing, (c) the right-side angel wing and the left side normal wing, (d) both-side angel wings.

The heavy body weight line, high egg production line, and their parent group were evaluated for the IAW and the types of AW based on appearance at 14 weeks of age. The types of AW observed included: both-sides normal wings, right-side AW with left-side normal wing, right-side normal wing with left-side AW and both-sides AWs. These four types of AW are illustrated in Figure 1.

### Threshold traits

Threshold characters mean these many characters of biological interest or economic importance which vary in a discontinuous manner but are not inherited in a simple Mendelian manner. Familiar examples are susceptibility to disease, where there are two phenotypic classes – affected or not-affected. When the underlying variable is below this threshold level the individual has one form of phenotypic expression, e.g. is ‘normal’; when it is above the threshold the individual has the other phenotypic expression, e.g. is ‘affected’ [22]. According Francis et al [5], AW is influenced by more than one gene. Besides, from on-site observation of the occurrence of AW in geese, we learned that AW mostly occurs during the growth period of geese (5–14 weeks old) and has two phenotypic expressions (normal and AW). Therefore, we thought AW occurs or not can be regarded as an all-or-none threshold character.

### Growth and reproduction performance

The performance evaluation of the heavy body weight line included measurements of birth weight, body weight at 8 and 14 weeks of age, body weight of the first-laying geese at the time of laying their first egg, the weight of the first egg, and the total number of eggs laid. For the high egg production line, in addition to evaluating the same traits as the heavy body weight line, the evaluation also included the body weight of the second-laying geese at the time of laying their first egg, the weight of the first egg, and the total number of eggs laid.

### Statistical analysis

The data collected were statistically analyzed using the general linear models procedure of SAS software [23]. For each variable measured, types of AW and sex were regarded as experimental treatments, respectively. That is:


(1) $${\text{Y}}_{\text{ijk}}=\mu +{\text{T}}_{\text{i}}+{\text{S}}_{\text{j}}+{\left(\text{T}\times \text{S}\right)}_{\text{ij}}+{ɛ}_{\text{ijk}}$$

Where Y_ijk_ = observed pen response; μ = overall mean; T_i_ = types of AW effect; S_j_ = sex effect; (L×S)_ij_ *=* random types of AW×sex effect; and ɛ_ijk_ = residual error.

The differences between the means of the two treatments were determined using the LSMEANS procedure with a statistical significance of p<0.05.

## RESULTS

### The types of the wings across generation

In the original G0 generation of the heavy body weight line, the types of wings were both-side normal wings (N type) accounted for 47.83% of the total population, the right-side normal wing (R type), the left side AW (L type) and both-side AWs (RL type) were 13.04%, 13.04% and 26.09%, respectively (Table 2). After six generations of selection for the heavy body weight line, in the G6 generation, N type was 44.31% of the population, while R type, L type, and LR type were 12.94%, 10.98%, and 31.76%, respectively.

The high egg production line originated from the same base population as the heavy body weight line. After three generations of selection for the high egg production line, in the G3 generation, N type was 61.82% of the population, while R type, L type, and LR type were 11.49%, 11.82%, and 14.86%, respectively (Table 2).

### Incidence of angel wing in population and offspring-parents

In the heavy body weight line of the White Roman population, there were a total of 990 geese, out of which 541 geese exhibited AW, resulting in an IAW of 54.6% (Table 3). The IAW was 65.6% of 294 progenies from families each comprised 1 and 4 angel-winged sire and dams, respectively. By consulting the table of truncated normal distribution for large sample, χ_P_, χ_R_ and i were obtained, and the values were −0.1156, −0.4014 and 0.7264, respectively. The estimate the liability heritability of AW incidence (LHIAW). LHIAWs of heavy body weight line was 0.39.

In the high egg production line of the White Roman population, there were a total of 775 geese, out of which 328 geese exhibited AW, resulting in an IAW of 42.3% (Table 3). The IAW was 43.5% of 124 progenies from families each comprised 1 and 4 angel-winged sire and dams, respectively. By consulting the table of truncated normal distribution for large sample, χ_P_, χ_R_ and i were obtained, and the values were 0.1942, 0.1635 and 0.9256, respectively (Table 4). The estimate the LHIAW. LHIAWs of high egg line was 0.03.

### Growth performance

The effects of the types of AW and sex on growth performances in the heavy body weight line of White Roman goose are mentioned in Table 5. There was no statistically significant interaction between wing types and sex. The types of the wings had no significant effect on body weight at birth in the heavy body weight line of White Roman goose (p>0.05). The RL type of geese was significantly heavier on body weight than those of N type and R type at 8 weeks old (4.19 vs 4.07 and 4.07 kg/bird; p = 0.0116). The male of geese was significantly heavier on body weight than that of female of geese at 8 weeks old (4.41 vs 3.81 kg/bird; p<0.0001). The male of geese was significantly heavier on body weight than that of female of geese at 14 weeks old (5.75 vs 4.88 kg/bird; p<0.0001).

The effects of the types of AW and sex on growth performances in the high egg production line of White Roman goose are mentioned in Table 6. There was no statistically significant interaction between wing types and sex. The types of the wings had no significant effect on body weight at birth in the high egg production line of White Roman goose (p>0.05). The N type of geese was significantly heavier on body weight than those of L type at 14 weeks old (4.86 vs 4.69 kg/bird; p = 0.0213). The male of geese was significantly heavier on body weight at birth than that of female of geese (103.3 vs 100.5 g/bird; p = 0.0157). The male of geese was significantly heavier on body weight than that of female of geese at 8 weeks old (4.00 vs 3.49 kg/bird; p<0.0001). The male of geese was significantly heavier on body weight than that of female of geese at 14 weeks old (5.12 vs 4.40 kg/bird; p<0.0001).

### Reproduction performance

The effects of the types of AW on reproductive performances in the heavy body weight line of White Roman goose are mentioned in Table 7. The types of the wings had no significant effect on body weight at first egg in the heavy body weight line of White Roman goose at first and second parity (p<0.05). The R type and RL type of geese was significantly heavier on egg weight at first egg than those of L type at first parity (147 and 146 vs 136 g/egg; p<0.05). The N type of geese was significantly heavier on egg weight at first egg than those of R type and RL type at first parity (174 vs 165 and 165 g/egg; p<0.05).

The effects of the types of AW on reproductive performances in the high egg production line of White Roman goose are mentioned in Table 8. The RL type of geese was significantly heavier on body weight than those of R type and L type at first parity (5.29 vs 4.80 and 4.98 kg/bird; p<0.05). The RL type of geese was significantly lighter on body weight than those of N type, R type and L type at first parity (5.04 vs 5.35, 5.42 and 5.39 kg/bird; p<0.05) The RL type of geese was significantly lighter on egg weight at first egg than those of hose of N type and R type at first parity (133 vs 139 and 143 g/egg; p<0.05).

## DISCUSSION

AW occurs during the growth period when the third and fourth metacarpal joints of the wing become misaligned, causing one or both sides of the primary feathers to twist outward [1,3,24]. AW mostly starts during the age of 6 to 14 weeks in White Roman geese in Taiwan [8]. In White Roman geese with AW, X-ray imaging has shown no abnormalities in skeletal structure [8,11]. However, the angle of the humerus and radial ulna in normal-winged geese is significantly greater than in those with AW [8,13]. A 64-slice computerized tomography scan revealed that AW is associated with a slight to moderate dilation in the carpometacarpal joint. Birds with AW have well-developed muscles [1], and there were no significant differences in the content of ash, calcium, and phosphorus in the bones [13] or the levels of copper, iron, manganese, and zinc in the blood biochemical parameters [8].

In Taiwan, the primary goose breeds are the White Roman goose and the Chinese goose, with the White Roman goose accounting for over 97%. The IAW in commercial geese ranges from 5% to 50%, with a higher incidence observed in Chinese geese compared to White Roman geese [11]. During slaughter, geese with AW require manual removal of primary feathers because the feathers tend to stick together. 70-day-old Hybrid-Wanxi geese, the IAW was found to be 8.86%, indicating that the probability of AW occurrence is influenced by breed rather than sex [13]. The progenies from parents with AW exhibited a higher IAW than those from parents with normal wings at both 8 and 14 weeks old in White Roman geese [11]. The IAW was 48.6% in the AW line and 14.8% in the commercial line of White Roman geese at 8 weeks old [14]. White Chinese geese suggested that AW is influenced by more than one gene, progenies from parents without AW had 85.3% normal wings and 14.7% AW, while progenies from parents with AW had 47.0% normal wings and 53.0% AWs [5]. In this study, the heavy body weight line and high egg production line on IAW were 54.6% (Table 3) and 42.3% (Table 4), which IAW were 65.6% and 43.5% from families each angel-winged sire and dams, respectively. This is consistent with the results of previous authors. The estimate the LHIAW. LHIAWs of heavy body weight line and high egg production line and were 0.39 and 0.03 (Tables 3, 4). In heavy body weight line on IAW and IAW from families each angel-winged sire and dams was high than high egg production line. A possible reason could be that the heavier body weight line was increased body weight is accompanied by a higher IAW. The selection for heavier body weight may have inadvertently led to a higher occurrence of this condition. Identified nine SNPs across five chromosomes that were significantly associated with the AW trait [7]. This suggests that selective breeding methods can be used to increase or decrease the incidence of IAW. Therefore, it is recommended that goose breeders implement independent culling to eliminate geese with AW, which may help reduce the incidence of this trait.

The sex had a significant effect on body weight at 6, 8, 10, 12, 14, and 16 weeks of age, with geese body weight increasing rapidly until 10–12 weeks of age, and only a slight increase after 12 weeks of age [25]. The male of the geese on boy weight, back length, sternum length, and chest girth were higher than that of the females during 13 to 25 weeks old [26]. In this study, the male was significantly heavier on body weight than that female in heavy body weight line and high egg production line at 8 and 14 weeks of age (Tables 5, 6). There was no statistically significant interaction between wing types and sex. This result is consistent with previous reports, where male geese generally grow faster than females. It also indicates that the occurrence of AW in geese does not significantly affect body weight across different sexes.

The RL type of geese in the heavy body weight line was significantly heavier at 8 weeks old compared to the N type and R type. However, by 14 weeks old, there was no significant difference in body weight among the different wing types (Table 5). In the high egg production line, the N type of geese was significantly heavier at 14 weeks old compared to the L type (Table 6). This indicates that the effect of wing type on body weight varies between the heavy body weight line and the high egg production line. In the process of selecting for the heavy body weight line, the different wing types did not affect the body weight of the geese, likely because selection was based on body weight at 8 and 14 weeks of age, leading to weight not differences among the wing types. However, in the selection process for the high egg production line, wing type does appear to influence body weight. The selection criteria focus on egg production rather than body weight. As a result, geese with AWs, which may be less competitive in intaking diet in group feeding, could have lighter body weights compared to normal-winged geese.

The egg-laying period decreased from 138 days in the 2nd parity to 117 days in the 4th parity in the White Koludzka breed, and the average weight of the eggs also decreased with each subsequent parity [27]. The heritability of body weight at 8 weeks of age for the W11 and W33 lines of White Koludzka geese as 0.64 and 0.76, respectively, and the heritability of body weight at 11 weeks of age as 0.50 and 0.46, respectively [28]. The heritability of body weight at 8 and 11 weeks of age for the Polish goose of white plumage was 0.64 and 0.68, with a genetic correlation of 0.92 between the two traits [29]. Estimated the heritability of body weight at 8 and 14 weeks of age for White Roman geese as 0.549 and 0.596, respectively, with a genetic correlation of 0.897 between these traits, indicating that body weight selection in geese is a highly heritable trait [30]. As a result, selecting for body weight can be highly effective. The egg production in Rhine geese increased by an average of 2.7 eggs per generation over six generations of selection for egg production [31]. The family selection for hatchability in Hungarian and Landaise geese over 15 years, there was an increase of one egg hatched per year and a 1% increase in egg fertilization rate [32]. Average egg weight from layers in their first laying season was 36% lower compared to goose eggs from the 4th production year [33]. The egg weight increases with the increasing age of the layer due to the positive correlation between egg weight and female body weight, and the high heritability (h^2^) of this trait, which is greater than 0.5 [34,35]. In this study, the first and second parities in the heavy body weight line (Table 7). However, there was a difference in body weight at first egg between the first and second parities in the high egg production line (Table 8). This difference may be related to the geese’s body weight and the feed intake during the resting period, as higher feed intake during this period could affect the female’s body weight. Additionally, the egg weight at first egg showed a statistically significant difference. Although egg weight is positively correlated with female body weight, in this study, the L type of geese had a lighter egg weight at first egg. The reason for this may be influenced by variations in feed intake.

The findings of IAW in White Roman geese revealed that only a few geese with slight AW of one wing or both wings during 6 to 12 weeks of age returned to NW during 10 to 14 weeks of age, possibly because the primary wings grow completely during 6 to 12 weeks of age [17]. The AW of masked boobies returned to NW at the fledging age [12]. The genetic selection for AW resulted in higher severity scores and an increased incidence of the condition in White Roman geese [14]. Conversely, feeding the birds a low-nutrition-concentration diet could reduce the IAW. The angles of the metacarpals and radioulnar bones in normal-winged geese tended to be larger than those in the AW group at 10 weeks of age. In terms of joint appearance, no significant differences were observed in the radiocarpal joints between normal and AW geese at 10 weeks old. However, in AW geese, the carpometacarpus was torqued outward away from the body laterally, with a slight to moderate dilation in the carpometacarpal joint. At 14 weeks of age, normal-winged geese exhibited a 9.24% greater angle compared to those with AW [8]. Based on previous research, AW has been shown to have a hereditary component. When selection is based on body weight, its heritability tends to be higher, suggesting a correlation between AW and body weight. A low-nutrition diet may help reduce its occurrence; however, the underlying genetic relationships warrant further investigation in future studies.

In this experiment, at 8 weeks of age, the RL type of geese in the heavy body weight line of White Roman geese was significantly heavier than the N type and R type. However, by 14 weeks of age, there was no difference in body weight among the different wing types. This phenomenon was not observed in the high egg production line of White Roman geese. The findings of IAW in White Roman geese were most evident between 6 and 12 weeks of age [17]. This may lead to the development of AW during the growth period, as the angles of the metacarpals and radioulnar bones in the AW group tended to be larger.

## CONCLUSION

In the heavy body weight line of the White Roman geese, the IAW was 54.6%. In the high egg production line of the White Roman geese, the IAW was 42.3%. The LHIAW in the heavy body weight line and the high egg production line were separately estimated as 0.39 and 0.03, respectively. The results imply that selection for heavy body weight may concomitantly select the gene of AWs.

## Figures and Tables

**Table 1:** Ingredients and nutrient compositions of the experimental diets (%, as fed basis)

Ingredients	Starter	Grower	Resting	Laying
Yellow corn	61.6	64.3	50.35	57.35
Soybean meal (44%)	29.0	21.5	12.7	25.7
Wheat bran	-	5.0	20.0	-
Rice bran	-	3.0	10.0	-
Alfalfa meal	-	-	-	2.0
Fish meal (65%)	3.5	-	-	2.5
Oyster shell	-	-	-	3.5
Cane molasses	3.0	3.0	3.0	3.0
Salt	0.3	0.3	0.3	0.3
Dicalcium phosphate	1.3	0.8	1.9	3.5
Limestone, pulverized	0.7	1.60	1.3	1.7
Choline chloride (50%)	0.1	0.1	0.1	0.1
DL-Methionine	0.25	0.2	0.1	0.15
Vitamin premix^1)^	0.1	0.1	0.1	0.1
Mineral premix^2)^	0.15	0.15	0.15	0.1
Total	100	100	100	100
Calculated nutrient values				
Crude protein (%)	20.0	15.5	13.0	18.0
ME (kcal/kg)	2900	2800	2350	2700
Total calcium (%)	0.82	0.73	1.02	3.11
Available phosphorus (%)	0.46	0.41	0.35	0.50

1) Diet was supplied per kilogram of diet: vitamin A, 10,000 IU; vitamin D3, 2,000 IU; vitamin E, 20 mg; vitamin K3, 1.5 mg; vitamin B1, 1.00 mg; vitamin B2, 4.8 mg; vitamin B6, 3.00 mg; vitamin B12, 16 μg; folic acid, 0.50 mg; calcium pantothenate, 10.0 mg; niacin, 25 mg; Biotin, 2.00 mg.

2) Diet was supplied per kilogram of diet: Fe (FeSO_4_), 120 mg; Cu (CuSO_4_.5H_2_O), 22.5 mg; Mn (MnSO_4_·H_2_O), 120 mg; Zn (ZnO), 75.0 mg; I (KI), 1.275 mg; Co (CoSO_4_), 0.375 mg; Se (Na_2_SeO_3_), 0.27 mg.

ME, metabolizable energy.

**Table 2:** The types of the wings across generations in the heavy body weight and high egg production line of White Roman Goose

Generation	The types of the wings				Total

	N	R	L	RL	
Heavy body weight line					
G0	33 (47.83)^1)^	9 (13.04)	9 (13.04)	18 (26.09)	69 (100)
G1	33 (63.46)	3 (5.77)	5 (9.62)	11 (21.15)	52 (100)
G2	66 (62.86)	16 (15.24)	8 (7.62)	15 (14.29)	105 (100)
G3	35 (41.18)	14 (16.47)	13 (15.29)	23 (27.06)	85 (100)
G4	50 (40.65)	8 (6.50)	18 (14.63)	47 (38.2^1)^	123 (100)
G5	119 (39.53)	45 (14.95)	31 (10.30)	106 (35.22)	301 (100)
G6	113 (44.3^1)^	33 (12.94)	28 (10.98)	81 (31.76)	255 (100)
G0–G6	449 (45.35)	128 (12.93)	112 (11.3^1)^	301 (30.40)	990 (100)
High egg production line					
G0	33 (47.83)	9 (13.04)	9 (13.04)	18 (26.09)	69 (100)
G1	129 (56.58)	18 (7.89)	15 (6.58)	66 (28.95)	228 (100)
G2	102 (56.04)	37 (20.33)	21 (11.54)	22 (12.09)	182 (100)
G3	183 (61.82)	34 (11.49)	35 (11.82)	44 (14.86)	296 (100)
G0–G3	447 (57.68)	98 (12.65)	80 (10.32)	150 (19.35)	775 (100)

1) Sample size and percentage of occurrence in each generation.

N, both-side normal wings; R, the right-side angel wing and the left side normal wing; L, the right-side normal wing and the left side angel wing; RL, both-side angel wings.

**Table 3:** Incidence of angel wing in population and offspring-parents of the heavy body weight line of White Roman goose

Item	Numbers		Incidence of angel wing		

	Angel wing	Total	p%	χ	i
P	541	990	54.6	−0.1156	0.7264
R	193	294	65.6	−0.4014	

P and R referred to the population and the offspring-parents, respectively.

χ, the normal deviate of the threshold from the mean, in standard-deviation units.

i, the mean deviation of angel-winged individuals.

**Table 4:** Incidence of angel wing in population and offspring-parents of the high egg production line of White Roman goose

Item	Numbers		Incidence of angel wing		

	Angel wing	Total	p%	χ	i
P	328	775	42.3	0.1942	0.9256
R	54	124	43.5	0.1635	

P and R referred to the population and the offspring-parents, respectively.

χ, the normal deviate of the threshold from the mean, in standard-deviation units.

i, the mean deviation of angel-winged individuals.

**Table 5:** Effects of the types of wing and sex on growth performance in the heavy body weight line of White Roman goose

Item	The types of the wings				Sex		TW	SEX	TW×SEX

	N	R	L	RL	Male	Female			
Body weight at birth (g/bird)	98.52±0.60^1)^ (447)^2)^	99.30±1.11 (128)	99.68±1.18 (11^2)^	101.12±0.74 (30^1)^	100.07±0.69 (484)	99.24±0.64 (504)	0.0590	0.3764	0.9279
Body weight at 8 weeks old (kg/bird)	4.07±0.02^b^ (44^2)^	4.07±0.04^b^ (125)	4.11±0.05^ab^ (11^1)^	4.19±0.03^a^ (30^1)^	4.41±0.03^x^ (478)	3.81±0.03^y^ (50^1)^	0.0116	<0.0001	0.7501
Body weight at 14 weeks old (kg/bird)	5.34±0.03 (40^2)^	5.22±0.05 (115)	5.35±0.06 (100)	5.36±0.04 (275)	5.75±0.03^x^ (45^2)^	4.88±0.03^y^ (440)	0.1818	<0.0001	0.6406

1) Mean±stander error of mean.

2) Figure in the parentheses was the sample size.

a,b Means without the same superscripts within the same row under the types of the wings treatment differ significantly (p<0.05).

x,y Means without the same superscripts within the same row under the sex treatment differ significantly (p<0.05).

N, both-side normal wings; R, the right-side angel wing and the left side normal wing; L, the right-side normal wing and the left side angel wing; RL, both-side angel wings; TW, The types of the wings.

**Table 6:** Effects of the types of wing and sex on growth performance in the high egg production line of White Roman goose

Item	The types of the wings				Sex		TW	SEX	TW×SEX

	N	R	L	RL	Male	Female			
Body weight at birth (g/bird)	101.55±0.58^1)^ (445)^2)^	101.31±1.30 (97)	101.71±1.43 (80)	103.0 3±1.08 (150)	103.28±0.92^x^ (336)	100.51±0.67^y^ (436)	0.6513	0.0157	0.9091
Body weight at 8 weeks old (kg/bird)	3.74±0.02 (430)	3.78±0.05 (93)	3.64±0.06 (79)	3.82±0.04 (149)	4.00±0.04^x^ (324)	3.49±0.03^y^ (427)	0.0869	<0.0001	0.1583
Body weight at 14 weeks old (kg/bird)	4.86±0.03^a^ (389)	4.73±0.06^ab^ (77)	4.69±0.06^b^ (66)	4.76±0.05^ab^ (13^1)^	5.12±0.04^x^ (29^2)^	4.40±0.03^y^ (37^1)^	0.0213	<0.0001	0.4183

1) Mean±stander error of mean.

2) Figure in the parentheses was the sample size.

a,b Means without the same superscripts within the same row under the types of the wings treatment differ significantly (p<0.05).

x,y Means without the same superscripts within the same row under the sex treatment differ significantly (p<0.05).

N, both-side normal wings; R, the right-side angel wing and the left side normal wing; L, the right-side normal wing and the left side angel wing; RL, both-side angel wings; TW, The types of the wings.

**Table 7:** Effects of the types of wing on reproductive performance in the line of heavy body weight of White Roman goose

Item	Treat				MSE

	N	R	L	RL	
The 1^st^ parity					
Body weight at first egg (kg)	5.30 (9^1)^^1)^	5.18 (4^1)^	5.18 (27)	5.28 (79)	0.40
Egg weight at first egg (g)	140^ab^ (90)	147^a^ (4^1)^	136^b^ (27)	146^a^ (79)	304
Egg (N)	21.6 (12^1)^	19.1 (5^1)^	18.6 (38)	20.6 (96)	196
The 2^nd^ parity					
Body weight at first egg (kg)	5.22 (33)	5.05 (16)	5.35 (7)	5.10 (2^1)^	0.22
Egg weight at first egg (g)	174^a^ (33)	165^b^ (16)	170^ab^ (7)	165^b^ (2^1)^	153
Egg (N)	25.4 (53)	21.6 (23)	28.0 (16)	26.3 (33)	333

1) Figure in the parentheses was the sample size.

a,b Means without the same superscripts within the same row under the types of the wings treatment differ significantly (p<0.05).

N, both-side normal wings; R, the right-side angel wing and the left side normal wing; L, the right-side normal wing and the left side angel wing; RL, both-side angel wings; MSE, mean square of error.

**Table 8:** Effects of the types of wing on reproductive performance in the line of high egg production of White Roman goose

Item	Treat				MSE

	N	R	L	RL	
The 1^st^ parity					
Body weight at first egg (kg)	5.21^ab^ (98)^1)^	4.80^c^ (29)	4.98^bc^ (25)	5.29^a^ (46)	0.37
Egg weight at first egg (g)	139^ab^ (96)	143^a^ (29)	137abc (25)	133^c^(45)	198
Egg (N)	22.2 (130)	19.2 (39)	23.5 (3^1)^	22.8 (63)	184
The 2^nd^ parity					
Body weight at first egg (kg)	5.35^a^ (7^1)^	5.42^a^ (27)	5.39^a^ (19)	5.04^b^ (24)	0.24
Egg weight at first egg (g)	164 (7^1)^	166 (27)	164 (19)	162 (24)	158
Egg (N)	29.6 (94)	26.9 (34)	34.2 (27)	25.6 (40)	275

1) Figure in the parentheses was the sample size.

a–c Means without the same superscripts within the same row under the types of the wings treatment differ significantly (p<0.05).

N, both-side normal wings; R, the right-side angel wing and the left side normal wing; L, the right-side normal wing and the left side angel wing; RL, both-side angel wings; MSE, mean square of error.
